# Synthesis of MBA-Encoded Silver/Silica Core-Shell Nanoparticles as Novel SERS Tags for Biosensing Gibberellin A_3_ Based on Au@Fe_3_O_4_ as Substrate

**DOI:** 10.3390/s19235152

**Published:** 2019-11-25

**Authors:** Qingmin Wei, Jianjuan Lin, Fa Liu, Changchun Wen, Na Li, Guobao Huang, Zhihui Luo

**Affiliations:** 1 Guangxi Key Laboratory of Agricultural Resources Chemistry and Biotechnology, Colleges and Universities Key Laboratory for Efficient Use of Agricultural Resources in the Southeast of Guangxi, College of Chemistry and Food Science, Yulin Normal University, Yulin 537000, China; weiqingmin09@163.com (Q.W.); L18877546716@163.com (J.L.); fa.l@163.com (F.L.); ln19860622@126.com (N.L.); 2 State Key Laboratory for Chemistry and Molecular Engineering of Medicinal Resources, School of Chemistry and Pharmaceutical Science, Guangxi Normal University, Guilin 541004, China; wcchun@163.com

**Keywords:** surface-enhanced Raman scattering, AgNPs@SiO_2_ core-shell nanoparticles, Au@Fe_3_O_4_ nanoparticle, gibberellin

## Abstract

A surface-enhanced Raman scattering (SERS) tag is proposed for high-sensitivity detection of gibberellin A_3_ (GA_3_). Silver nanoparticles (AgNPs) were synthesized using citrate reduction. 4-Mercaptobenzoic acid (MBA) was used for the Raman-labeled molecules, which were coupled to the surface of the AgNPs using sulfydryls. MBA was coated with silica using the Stöber method to prevent leakage. GA_3_ antibodies were attached via the active functional groups N-Hydroxysuccinimide (NHS) and N-Ethyl-N’-(3-dimethylaminopropyl)carbodiimide hydrochloride (EDC) to construct a novel immuno-AgNPs@SiO_2_ SERS tags. The captured SERS substrates were fabricated through Fe_3_O_4_ nanoparticles and gold nanoparticles (AuNPs) using chemical methods. These nanoparticles were characterized using ultraviolet-visible spectroscopy (UV–Vis), dynamic light scattering, Raman spectroscopy, transmission electron microscope (TEM), and X-ray diffraction (XRD). This immuno-AgNPs@SiO_2_ SERS tags has a strong SERS signal based on characterizations via Raman spectroscopy. Based on antigen-antibody reaction, the immuno-Au@Fe_3_O_4_ nanoparticles can capture the GA_3_ and AgNPs@SiO_2_ SERS tags. Due to the increasing number of captured nanoprobes, the SERS signal from MBA was greatly enhanced, which favored the sensitive detection of GA_3_. The linear equation for the SERS signal was y = −13635x + 202211 (R^2^ = 0.9867), and the limit of detection (LOD) was 10^−10^ M. The proposed SERS tags are also applicable for the detection of other food risk factors.

## 1. Introduction

Gibberellins (GAs) is a type of extensive plant growth regulator that promotes plant cell division, enhances plant growth and development, prolongs the preservation and freshness storage of fruit, and increases the output of seedless grapes [1,2]. Up to now, more than a hundred gibberellins have been isolated and determined in plants, fungi, and bacteria. Despite their structural similarity, only a small number of GAs display biological activity in plants, such as GA_3_, GA_1_, GA_7_, and GA_4_. Most of the GAs are considered to be precursors, intermediates and metabolites [3,4]. As is true for most pesticides, GAs may be harmful to humans who receive excessive exposure. Although such damage may not be developed over a short period, there is residual GAs in many fruit and vegetables. Excessive ingestion of GAs may cause damage to the normal internal secretory system, chronic organ toxicity, and cancer [5,6,7]. There are several existing methods for GAs detection, including high-performance liquid chromatography (HPLC) [4], gas chromatography-mass spectrometry [8], liquid chromatography–electrospray tandem mass spectrometry (LC-MS) [9], and others [10]. However, liquid extraction and thin layer separation are needed for sample processing, which is complicated, time-consuming, and requires the abundant use of organic solvents.

SERS is a smart spectrum technology that has been developed over the past three decades. SERS can provide non-destructive and ultra-sensitive detection at the single molecule level, which is comparable with monomolecular fluorescence spectroscopy [11,12,13]. Due to the advantages of SERS—high sensitivity, high selectivity, and non-destructiveness—it has become a common tool in chemistry, biomedicine, and physics. It has been widely applied in many fields, including drug development, food safety, disease detection, explosives detection, imaging, minerals, and archaeology [14,15,16,17]. 

Immunoreactions provide a reliable, simple, and cheap method to recognize and perform quantitative analyses on specific antibodies or antigens. Therefore, several works have combined these two methods to construct a surface-enhanced Raman-immune detection system that has been extensively used to study small biological molecules, pathogenic bacteria, cells, and live imaging [18,19,20].

**Scheme 1:**
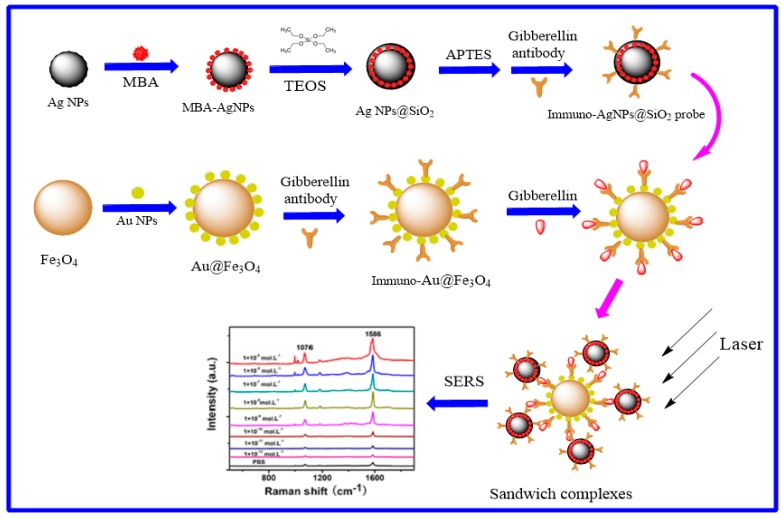
Schematic representation of the synthesis procedure of the AgNPs@SiO_2_ SERS tags for the determination of GA_3_.

Herein, a quick detect, non-separated, high sensitivity, and cheap surface Raman-immuno detection system has been developed for the determination of GA_3_. Scheme 1 describes the fabrication process of the AgNPs@SiO_2_ SERS tags for GA_3_. The Raman reporter MBA were coupled on the surface of the AgNPs, and then were coated with SiO_2_ using the Stöber method to prevent leakage. GA_3_ antibodies were connected via active functional groups of NHS and EDC after amination of AgNPs@SiO_2_, and a novel immuno-AgNPs@SiO_2_ SERS tags was obtained. The captured SERS substrates (immuno-Au@Fe_3_O_4_ nanoparticles) were fabricated through Fe_3_O_4_ nanoparticles and AuNPs using chemical methods. Based on antigen-antibody reaction, immuno- Au@Fe_3_O_4_ nanoparticles can be captured the GA_3_ and AgNPs@SiO_2_ SERS tags. Due to the increasing number of captured nanoprobes, the SERS signal from MBA was greatly enhanced which favored the sensitive detection of GA_3_. The results suggested that the method possessed excellent potential for diagnostic immunoassay.

## 2. Materials and Methods 

### 2.1. Reagents and Materials 

Silver nitrate, trisodium citrate, chloroauric acid, ferric chloride, sodium acetate, polyethylene glycol, bovine serum albumin (BSA), isopropanol, and absolute ethylalcohol were obtained from Shanghai Sinopharm Chemical Regent Co. Ltd. Gibberellin A_3_ (GA_3_), forchlorfenuron, and the gibberellin antibody were purchased from Shanghai Yuanye biology Co. Ltd. 4-Mercaptobenzoic acid (MBA), Tetraethyl orthosilicate (TEOS), 3-aminopropyl-trimethylsilane (APTES), N-Hydroxysuccinimide (NHS), and N-Ethyl-N’-(3-dimethylaminopropyl)carbodiimide hydrochloride (EDC) were purchased from Aladdin Chemicals Co. Ltd (Shanghai, China). For all aqueous solution, high-purity ultrapure water (18.18 MΩ cm) from a Millipore system was used for all experiments.

### 2.2. Measurements

All nanoparticles were characterized using transmission electron microscopy (TEM, Hitachi, Japan), scanning electron microscope (SEM, Hitachi, Japan), and X-ray diffraction (XRD, Rigaku, Japan). UV–Vis absorption spectra were acquired on a Cary 5000 spectrophotometer (Agilent, USA) with a 1.00 cm quartz cuvette sample holder. SERS were obtained using a Raman spectrometer 2000 (Renishaw, UK) equipped with a confocal microscope (Leica, Germany). A He-Ne laser (785 nm) excitation source was used in the experiment. A 20× objective lens was used to focus a laser spot on materials with a power of approximately 1 mW. The Raman spectra were collected with a 10 s exposure time and 2 accumulations from 400–2000 cm^−1^.

### 2.3. Synthesis of Ag Nanoparticles 

AgNPs were synthesized by dissolving 0.018 g of silver nitrate in 100 mL of ultrapure water. The mixture was slowly heated to 50 °C, after which 2 mL of 1% trisodium citrate solution was quickly added, and the solution was brought to a boil for 1 h. The solution was then cooled to room temperature. The obtained AgNPs were filtered with a 0.22 μm film and kept at 4 °C. 

### 2.4. Synthesis of AgNPs@SiO_2_ Core-Shell Nanoparticles

An 8 mL AgNPs was mixed with 40 μL of 10^−3^ M MBA and allowed to react for 30 min at room temperature. The mixture was centrifuged for 5 min at 9000 rpm and resuspended in 2 mL of ultrapure water. Then, 10 mL of isopropanol was added to achieve an alcohol-water ratio of 5:1. The pH was adjusted to ~ 9.5 using ammonium hydroxide. Then, 30 μL of TEOS was reacted with the nanoparticles for 30 min. The mixture was centrifuged for 5 min at 8000 rpm and rinsed twice using absolute ethyl alcohol. The prepared samples were then retained for later use.

### 2.5. Construction of Immuno-Ag@SiO_2_ Core-Shell SERS Tags

A 4 mL Ag@SiO_2_ core-shell nanoparticles and 40 μL APTES were mixed and reacted at room temperature for 30 min. The mixture was centrifuged for 5 min at 8000 rpm and resuspended in 2 mL of phosphate buffer saline (PBS). After, 100 μL of GA_3_ antibody, conjugated to NHS and EDC, was added in core-shell nanoparticles and reacted for 60 min. Next, 200 μL of 1% BSA was added to block excess binding sites on the core-shell nanoparticles. The nanoparticles were centrifuged for 5 min at 8000 rpm and rinsed twice with PBS to obtain the core-shell SERS tags. The prepared core-shell SERS tags were retained for later use. 

### 2.6. Characterized of the Obtained Nanoparticles by SEM and TEM

For SEM and TEM characterization, 2 μL of the obtained nanoparticles (Ag@SiO_2_ and Au@Fe_3_O_4_) were put on a copper grid and dried at the room temperature. They were then transferred to detect on the test platform and exhausted under vacuum. The morphology of nanoparticles was observed under 200 KV.

### 2.7. Synthesis of Fe_3_O_4_ Nanoparticles 

Fe_3_O_4_ nanoparticles were synthesized according li group method [21]. 0.68 g of ferric chloride was dissolved in ethylene glycol (20 mL), followed by the addition of sodium acetate (1.8 g) and polyethylene glycol (0.5 g) under stirring vigorously, and transferred to a 25 mL Teflon lined stainless-steel autoclave. The autoclave was heated to and maintained at 200 °C for 7 h, and allowed to cool to room temperature. The black products were washed four times with ethanol and dried at 60 °C for 5 h.

A collection of 200 nm Fe_3_O_4_ nanoparticles (270 mg) were dispersed in 30 mL of methylbenzene and poured into a round-bottom flask. Then, 0.7 mL APTES was added and the mixture was stirred at 250 rpm at 80 °C for 8 h reflux. The reactants were rinsed with ultrapure water and ethyl alcohol alternatively until obtaining a neutral water solution. Impurities on the product surface were eliminated and the amino-Fe_3_O_4_ nanoparticles were stored in 4 °C ethyl alcohol for later use.

### 2.8. Synthesis of Au@Fe_3_O_4_ Nanoparticles 

The dried, modified, amino-Fe_3_O_4_ powder (8 mg) and 110 mL of ultrapure water were mixed. After ultrasonic dispersion, 15 mL of 1.2 mmol chloroauric acid was added to the mixture and stirred evenly. Then, 570 μL of 0.2 M sodium citrate solution was added under ultrasonic conditions. Given a fixed frequency, the mixture was processed using ultrasonic processing at room temperature until the solution changed color from light yellow to black. At the end of the reaction, a permanent magnet was used for solution separation, which was cleaned with pure water three times until reaching a neutral pH.

### 2.9. Assembly of Immuno-Au@Fe_3_O_4_ Nanoparticles 

A 10 mL sample of 0.5 mg/mL the Au@Fe_3_O_4_ nanoparticles was collected, and the pH was adjusted to 8.0. Then, 10 μL of 1 mg/mL GA_3_ antibody was added and stirred via rotation for 0.5 h at room temperature. A 1.1 mL solution of 10% BSA was added to block the excess sites on the surface of Au@Fe_3_O_4_ nanoparticles. After centrifugation and dispersal, the immuno-Au@Fe_3_O_4_ nanoparticles were obtained and keep at 4 °C. 

### 2.10. Immunoassay Detection of GA_3_ Based on SERS Tags and Magnetic Nanoparticles

Ten pieces of eppendorf tubes were added to 50 μL of 0.1 mg/mL the immuno-Au@Fe_3_O_4_ nanoparticles. A series of different concentrations of GA_3_, in 100 μL, was then added and reacted for 2 h at room temperature. After magnetic separation, 100 μL of PBS was added to disperse the sample. Next, 40 μL of the SERS tags and 100 μL of PBS were added and reacted for 2 h at room temperature. After performing magnetic separation twice, the sandwich complexes were formed and used for SERS detection.

## 3. Results and Discussion

### 3.1. UV–Vis Spectroscopy of AgNPs, MBA-AgNPs, Ag@SiO_2_ Core-Shell Nanoparticles

**Figure 1:**
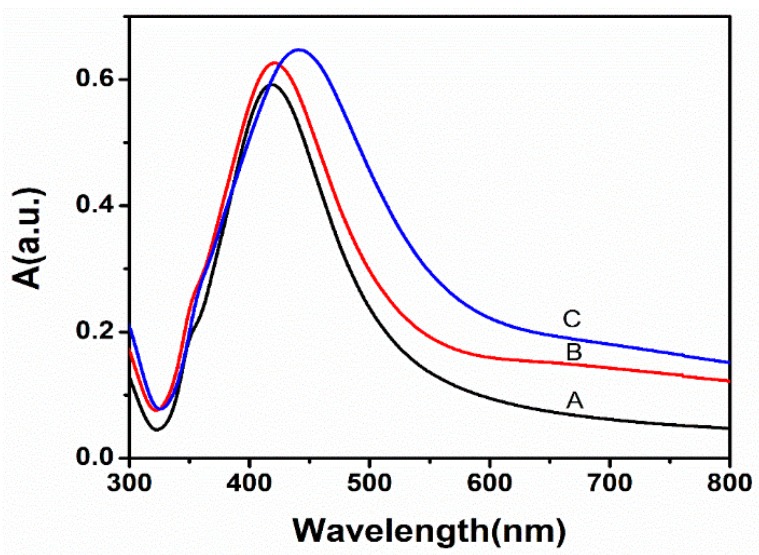
UV–Vis spectroscopy of AgNPs (**A**), MBA-AgNPs (**B**), and Ag@SiO_2_ core-shell nanoparticles (**C**).

To verify whether the Ag@SiO_2_ core-shell nanoparticles were successfully synthesized, the obtained nanoparticles were characterized via UV–Vis spectroscopy, as illustrate in Figure 1. The maximum UV–Vis absorbance peak for the AgNPs was observed at 419 nm, which agrees with previous studies [22]. The peak width at half height is narrow and the peak shape is relatively symmetric, which indicates that the synthesized AgNPs have a relatively uniform shape and size. After an appropriate amount of MBA was added, the maximum absorbance peak of the solutions shifted to 420 nm, while the peak width at half height increased slightly. This indicates that the MBA was successfully coupled onto the AgNPs. The maximum absorbance peak of the obtained nanoparticles shifted from 419 to 460 nm when silica was forming. It is very well known effect and from a simple model of the plasmon resonance in metal nanoparticles one can expect that coating with the material with the higher refractive induces the red-shift of the plasmon bands of metal nanoparticles [23,24]. 

### 3.2. TEM of AgNPs and Ag@SiO_2_ Core-Shell Nanoparticles 

**Figure 2:**
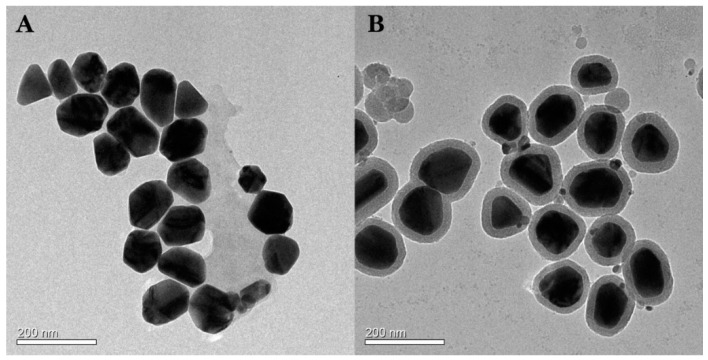
TEM of AgNPs (**A**) and Ag@SiO_2_ core-shell nanoparticles (**B**).

**Figure 3:**
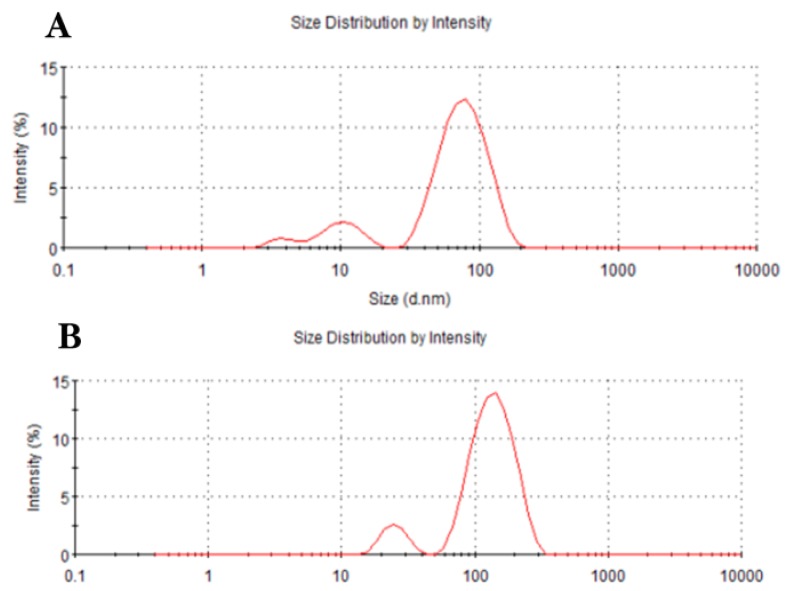
Dynamic light scattering of AgNPs(**A**) and Ag@SiO_2_ core-shell nanoparticles (**B**).

To determine whether the synthesized nanoparticles have a core-shell structure and an appropriate size, their morphology was characterized using TEM. The TEM micrographs of the AgNPs and Ag@SiO_2_ nanoparticles are shown in Figure 2. The synthesized AgNPs were well-dispersed and without aggregation from Figure 2A. The particle size was approximately 90 nm. After the silicon was coupled onto the AgNPs, the particle size increased. The particles have a clear core-shell structure, and the thickness of the surface silicon layer is around 20 nm from Figure 2B. Dynamic light scattering was used to confirm our result, as shown in Figure 3, zeta potential of AgNPs and Ag@SiO_2_ core-shell nanoparticles is −18.5 mV and −15.4 mV because of reducing citrate on the surface of AgNPs, and the hydrodynamic diameter of Ag@SiO_2_ was about 140 nm, while the hydrodynamic diameter of AgNPs is 95 nm.

### 3.3. UV–Vis Spectroscopy of Fe_3_O_4_ and Au@Fe_3_O_4_ Nanoparticles

**Figure 4:**
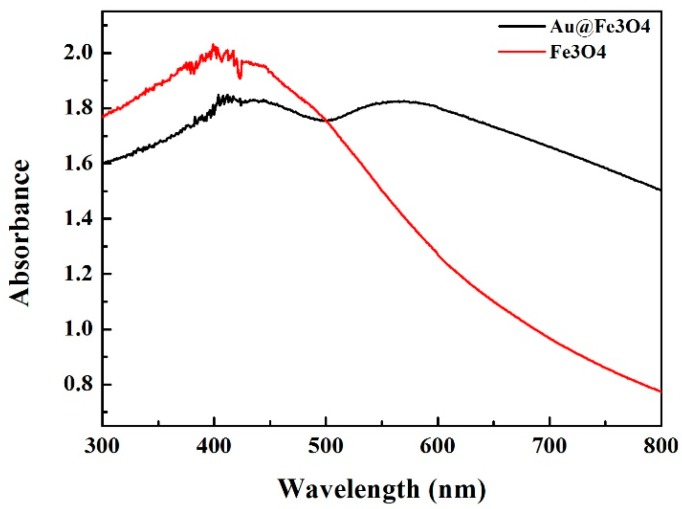
UV–Vis spectroscopy of Fe_3_O_4_ and Au@Fe_3_O_4_ nanoparticles.

Synthesis of the Au@Fe_3_O_4_ nanoparticles was preliminarily monitored using UV–Vis spectroscopy for Fe_3_O_4_ nanoparticles and Au@Fe_3_O_4_ nanoparticles, as shown in Figure 4. The Au@Fe_3_O_4_ nanoparticles have a maximum absorbance peak at 560 nm, while the Fe_3_O_4_ nanoparticles have no such peak. This suggests that gold enveloped the magnetic nanoparticles.

### 3.4. Dynamic Light Scattering of Fe_3_O_4_ and Au@Fe_3_O_4_ Nanoparticles

**Figure 5:**
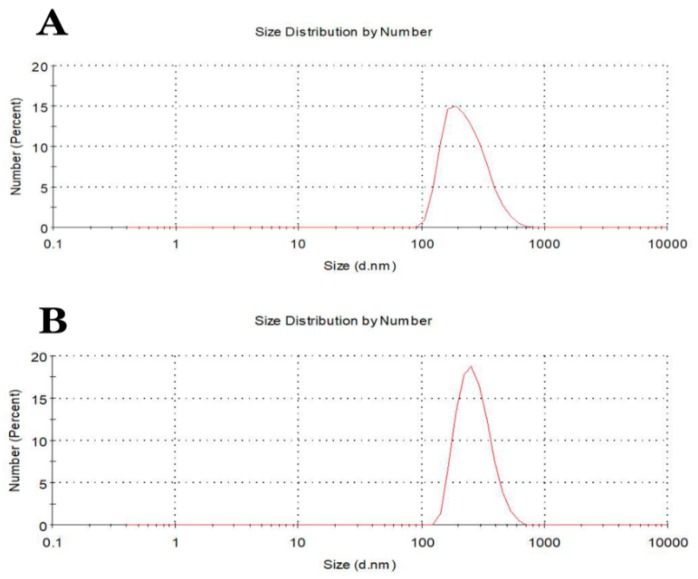
Dynamic light scattering of Fe_3_O_4_ (**A**) and Au@Fe_3_O_4_ nanoparticles(**B**).

During the synthesis of the Au@Fe_3_O_4_ nanoparticles, the intermediate products of each step were monitored using a dynamic light scattering instrument. The Fe atoms, which are exposed on the Fe_3_O_4_ surface, easily adsorb the OH^−^ in water to form -OH functional groups. These functional groups have negative potentials as indicated by the increase in Zeta potential from −35.1 to −8.87 mV. The potential increased, indicating that the -NH_2_ and -OH on the magnetic nanoparticles react to form amino functional groups. As shown in Figure 5, the hydrodynamic diameter of the amino- Fe_3_O_4_ was 237.4 nm in Figure 5A. When the Zeta potential increased to 30.6 mV, the magnetic nanoparticles were completely enveloped with gold to form Au@Fe_3_O_4_, the hydrodynamic diameter also increased to 275 nm in Figure 5B.

### 3.5. X-Ray Diffraction Characterization of Au@Fe_3_O_4_ Nanoparticles

**Figure 6:**
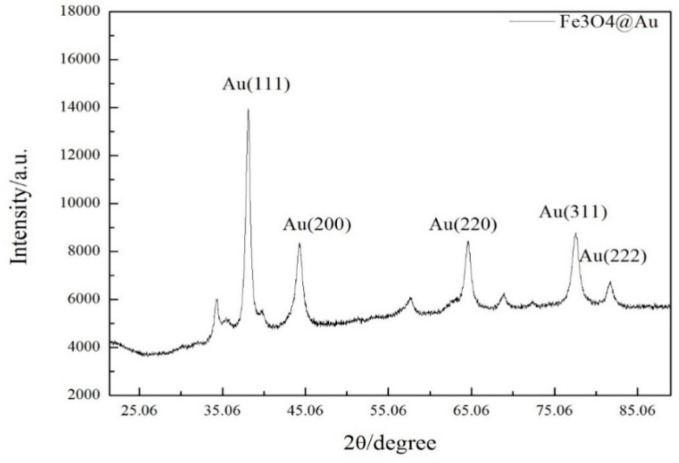
X-ray diffraction characterization of Au@Fe_3_O_4_ nanoparticles.

X-ray diffraction measurements were performed to determine the structure and morphology of the internal atoms and molecules of the materials. The characteristic peaks of gold were observed at 111, 200, 220, 311, and 222, indicating that the magnetic coating on the samples was made of elemental gold (Figure 6).

### 3.6. SEM Characterization of Fe_3_O_4_ and Au@Fe_3_O_4_ Nanoparticles

**Figure 7:**
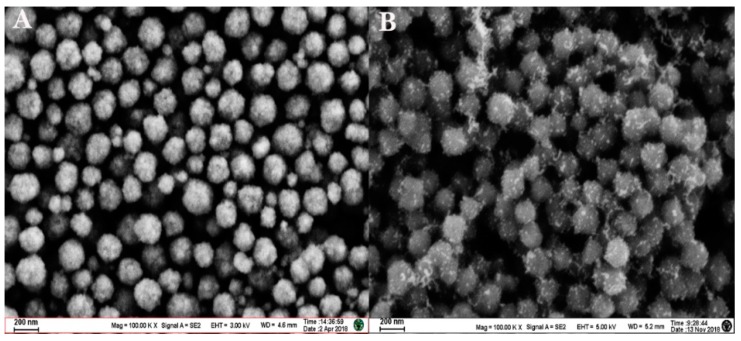
SEM characterization of Fe_3_O_4_ (**A**) and Au@Fe_3_O_4_ nanoparticles (**B**).

To determine the optimal synthesis conditions of the Au@Fe_3_O_4_ nanoparticles, the morphologies for the Fe_3_O_4_ and Au@Fe_3_O_4_ nanoparticles were compared under SEM. Figure 7 shows micrographs for (A) the 200 nm magnetic nanoparticles and (B) the Au@Fe_3_O_4_ nanoparticles. The magnetic nanoparticles have smooth surfaces while the Au@Fe_3_O_4_ surfaces are rough, which is caused by gold aggregation. These results are consistent with the zeta potential and XRD spectra. Therefore, gold is coated around the magnetic nanoparticle surface.

### 3.7. Raman Spectra of Solid MBA and AgNPs@SiO_2_ SERS Tags 

**Figure 8:**
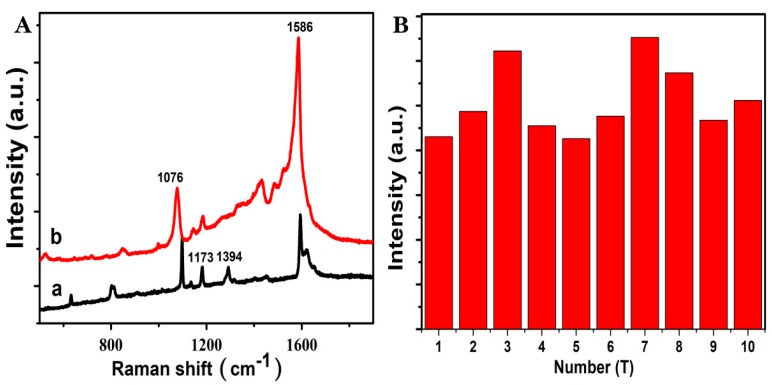
The Raman spectra of solid MBA and the AgNPs@SiO_2_ SERS probe (**A**). The SERS intensity of the AgNPs@SiO_2_ tags at the 1586 cm^−1^ peak of MBA (**B**).

To further determine whether the synthesized AgNPs@SiO_2_ tags show SERS activity, a Raman test on solid MBA and the probes was performed. The Raman spectra were obtained using the 785 nm laser, as shown in Figure 8A, the synthesized SERS probe shows a stronger Raman response compared to the Raman spectra of solid MBA. Peaks at 1076, 1173, 1394, and 1586 cm^−1^ correspond to the fingerprints of the MBA. The two Raman peaks at 1076 and 1586 cm^−1^ are attributed to the v_12_ and v_8a_ vibrations of the benzene ring on MBA [25,26]. The peak width at half height at 1586 cm^−1^ is relatively small and can be used to perform a quantitative analysis. For evaluating the SERS tags reproducibility, we measured SERS signals of MBA at 10 randomly chosen spots on the substrate, the relative standard deviation of the intensity at the 1586 cm^−1^ peak of MBA was calculated to be 15.55% (Figure 8B). The results indicated that the SERS tags had good reproducibility and stability during the preparation.

### 3.8. SERS Detection of GA_3_

**Figure 9:**
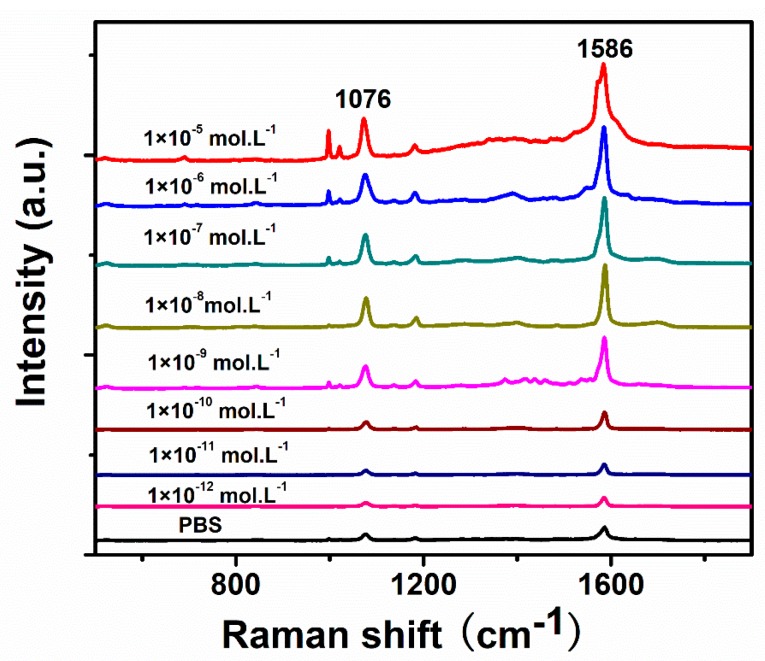
SERS spectra for decreasing concentrations of GA_3_.

**Figure 10:**
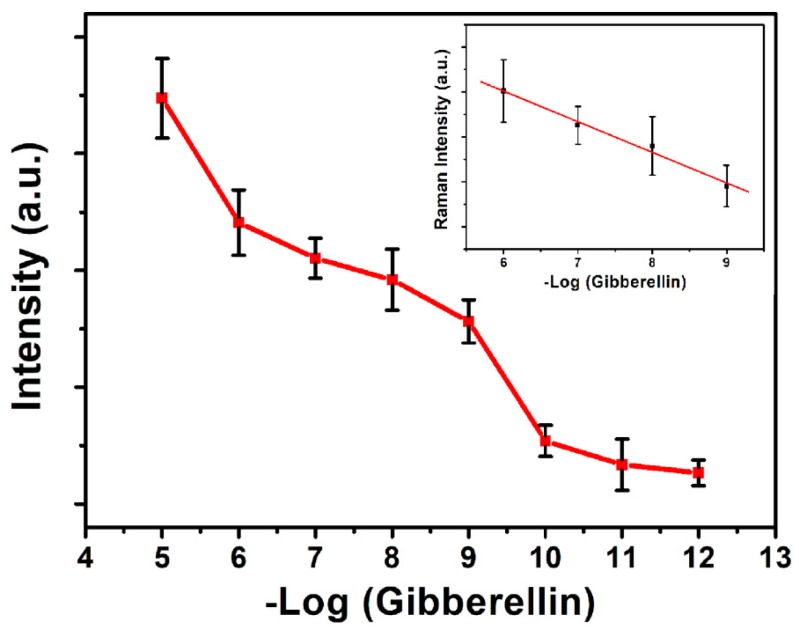
Plot ratios of SERS intensities with the sample versus a series concentration of GA_3_. Insert: the linear relationship of SERS intensity versus GA_3_ concentration from 1 × 10^−6^ to 1 × 10^−9^ M, the error bars indicate standard deviations from three measurements.

The synthesized SERS tags was used to detect GA_3_. First, the immuno-Au@Fe_3_O_4_ nanoparticles captured GA_3_ through antigen-antibody reactions. Second, the immuno-SERS probe was recognized automatically using the immunomagnetic nanoparticles to establish a sandwich detection model. The analytical performance was assessed by measuring the SERS intensity at 1586 cm^−1^ of MBA as a function of the GA_3_ concentration using the 785 nm laser. There is a positive correlation between the concentration of GA_3_ and the Raman signal intensity of MBA. Figure 9 shows the SERS spectra for a concentration series of GA_3_. In the absence of GA_3_, a weak SERS signal was obtained (which was used as a blank), indicating that a small amount of SERS probe remained on the Fe_3_O_4_ after magnetic separating. When GA_3_ was added, the SERS intensity at 1586 cm^−1^ obviously increased. From the Figure 10, a linear regression equation, y = −13,635x + 202,211 (R^2^ = 0.9867) was obtained between the negative logarithm of the concentration of GA_3_, with a LOD of 10^−10^ M, where y is the intensity of SERS peak at 1586 cm^−1^ and x is the negative logarithm of GA3 concentration. This suggests that the proposed method is applicable for GA_3_ detection. Compared to other methods, such as HPLC coupled with fluorescence detection [4], UPLC-MS [3,27], the proposed method had not only equivalent to sensitivity, but also had advantage of fast detection, free-separated.

## 4. Conclusions

Based on the facile separation of magnetic nanoparticles, a new method to detect GA_3_ was developed based on SERS tags. SERS tags were constructed using AgNPs as a SERS substrate with embedded MBA by coating SiO_2_. Au@Fe_3_O_4_ nanoparticles played a separation role, and SERS enhanced the functional role. Quantitative analysis of GA_3_ was conducted using the SERS tags. The detection sensitivity was found to be 10^−10^ M. Compared with traditional detection methods, the developed method is fast, free-separated, and high sensitivity. It is a method that can be generalized and applied to other large and small single-biological molecules.

## References

[1] Olszewski N., Sun T.-P., Gubler F. (2002). Gibberellin signaling: biosynthesis, catabolism, and response pathways. The Plant Cell.

[2] Hedden P., Thomas S.G. (2012). Gibberellin biosynthesis and its regulation. Biochem. J..

[3] Manzi M., Gómez-Cadenas A., Arbona V. (2015). Rapid and reproducible determination of active gibberellins in citrus tissues by UPLC/ESI-MS/MS. Plant Physiol. Biochem..

[4] Lu Y.-H., Cao Y.-M., Guo X.-F., Wang H., Zhang H.-S. (2016). Determination of gibberellins using HPLC coupled with fluorescence detection. Anal. Methods.

[5] El-Mofty M., Sakr S., Rizk A., Moussa E. (1994). Carcinogenic effect of gibberellin A3 in Swiss albino mice. Nutr. Cancer.

[6] El-Mofty M., Sakr S. (1988). Induction of neoplasms in the Egyptian toad Bufo regularis by gibberellin A3. Oncology.

[7] Waqas M., Khan A.L., Kamran M., Hamayun M., Kang S.-M., Kim Y.-H., Lee I.-J. (2012). Endophytic Fungi Produce Gibberellins and Indoleacetic Acid and Promotes Host-Plant Growth during Stress. Molecules.

[8] Nehela Y., Hijaz F., Elzaawely A.A., El-Zahaby H.M., Killiny N. (2016). Phytohormone profiling of the sweet orange (Citrus sinensis (L.) Osbeck) leaves and roots using GC–MS-based method. J. Plant Physiol..

[9] Hao Y.-H., Zhang Z., Wang L., Liu C., Lei A.-W., Yuan B.-F., Feng Y.-Q. (2015). Stable isotope labeling assisted liquid chromatography–electrospray tandem mass spectrometry for quantitative analysis of endogenous gibberellins. Talanta.

[10] Pan C., Tan S.N., Yong J.W., Ge L. (2017). Progress and development of analytical methods for gibberellins. J. Sep. Sci..

[11] Wang A.X., Kong X. (2015). Review of recent progress of plasmonic materials and nano-structures for surface-enhanced Raman scattering. Materials.

[12] Kneipp K., Wang Y., Kneipp H., Perelman L.T., Itzkan I., Dasari R., Feld M.S. (1997). Single molecule detection using surface-enhanced Raman scattering (SERS). Phys. Rev. Lett..

[13] Qian X.M., Peng X.H., Ansari D.O., Yin-Goen Q., Chen G.Z., Shin D.M., Yang L., Young A.N., Wang M.D., Nie S.M. (2008). In vivo tumor targeting and spectroscopic detection with surface-enhanced Raman nanoparticle tags. Nat. Biotechnol..

[14] Dies H., Raveendran J., Escobedo C., Docoslis A. (2018). Rapid identification and quantification of illicit drugs on nanodendritic surface-enhanced Raman scattering substrates. Sens. Actuators, B Chem..

[15] Alvarez-Puebla R.A., Liz-Marzan L.M. (2012). Traps and cages for universal SERS detection. Chem. Soc. Rev..

[16] Pieczonka N.P.W., Aroca R.F. (2008). Single molecule analysis by surfaced-enhanced Raman scattering. Chem. Soc. Rev..

[17] Huang Y., Lin D., Li M., Yin D., Wang S., Wang J. (2019). Ag@Au Core–Shell Porous Nanocages with Outstanding SERS Activity for Highly Sensitive SERS Immunoassay. Sensors.

[18] Porter M.D., Lipert R.J., Siperko L.M., Wang G., Narayanana R. (2008). SERS as a bioassay platform: Fundamentals, design, and applications. Chem. Soc. Rev..

[19] Li J.F., Zhang Y.J., Ding S.Y., Panneerselvam R., Tian Z.Q. (2017). Core-Shell Nanoparticle-Enhanced Raman Spectroscopy. Chem. Rev..

[20] Kim K., Choi N., Jeon J.H., Rhie G.-E., Choo J. (2019). SERS-Based Immunoassays for the Detection of Botulinum Toxins A and B Using Magnetic Beads. Sensors.

[21] Deng H., Li X., Peng Q., Wang X., Chen J., Li Y. (2005). Monodisperse Magnetic Single-Crystal Ferrite Microspheres. Angew. Chem. Int. Ed..

[22] Zhang X., Kong X., Lv Z., Zhou S., Du X. (2013). Bifunctional quantum dot-decorated Ag@SiO2 nanostructures for simultaneous immunoassays of surface-enhanced Raman scattering (SERS) and surface-enhanced fluorescence (SEF). J. Mater. Chem. B.

[23] Lu Y., Yin Y., Li Z.-Y., Xia Y. (2002). Synthesis and Self-Assembly of Au@SiO2 Core−Shell Colloids. Nano Lett..

[24] Abdulrahman H.B., Krajczewski J., Aleksandrowska D., Kudelski A. (2015). Silica-Protected Hollow Silver and Gold Nanoparticles: New Material for Raman Analysis of Surfaces. J. Phys. Chem. C.

[25] Orendorff C.J., Gole A., Sau T.K., Murphy C.J. (2005). Surface-Enhanced Raman Spectroscopy of Self-Assembled Monolayers:  Sandwich Architecture and Nanoparticle Shape Dependence. Anal. Chem..

[26] Luo Z., Li W., Lu D., Chen K., He Q., Han H., Zou M. (2013). A SERS-based immunoassay for porcine circovirus type 2 using multi-branched gold nanoparticles. Microchim. Acta.

[27] Yan Z., Nie J., Xu G., Li H., Li J., Li Z., Wu Y., Kuang L. (2016). Simultaneous Determination of Plant Growth Regulators in Fruits Using a Modified QuEChERS Procedure and UPLC–MS/MS. Horticultural Plant J..

